# Supplementary material for: Contextual Hospital Conditions and the Risk of Nosocomial SARS-CoV-2 Infection: A Matched Case-Control Study with Density Sampling in a Large Portuguese Hospital
Source: J Clin Med. 2024 Sep 5;13(17):5251. doi: 10.3390/jcm13175251 (PMC11396589; doi:10.3390/jcm13175251)
Supplement: Supplementary file 1 [file jcm-13-05251-s001.zip › jcm-3137664-supplementary.pdf]

Figure S1 - Controls selection criteria

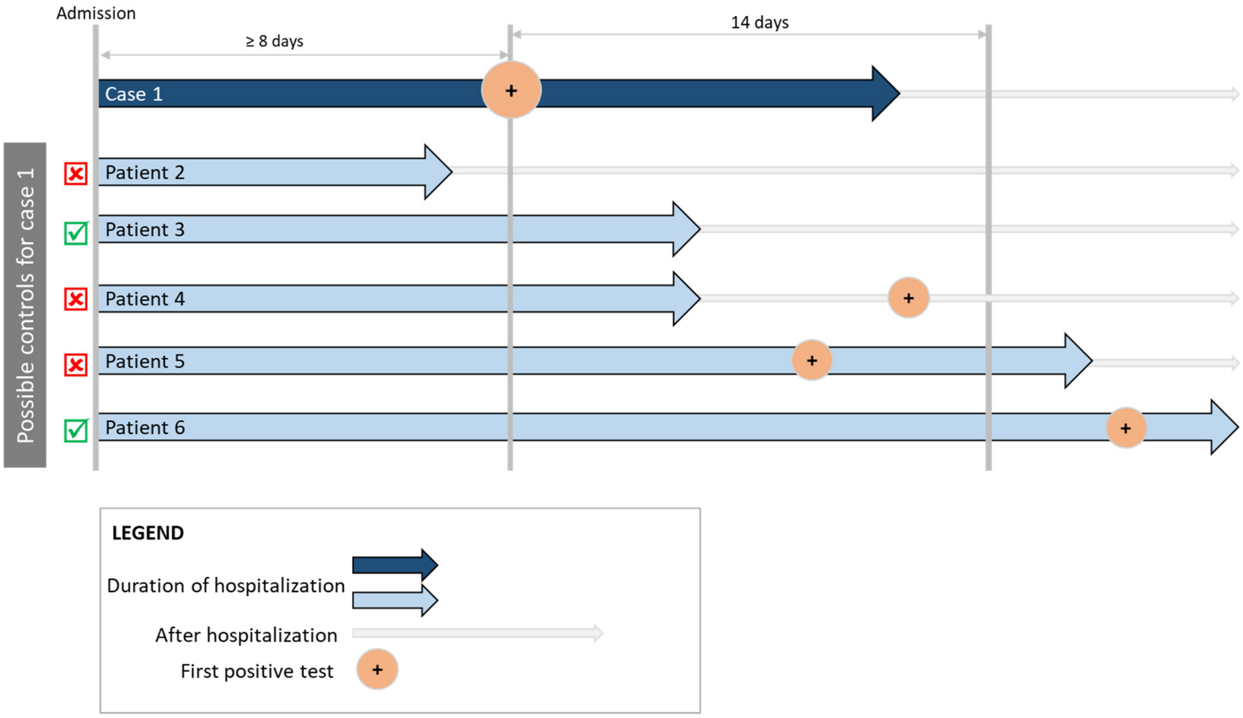

Patients 5 and 6 are also eligible cases

Table S1 – List of clinical procedures with risk of generating aerosols

Clinical procedures	*Area of health record registration*	*Designation*
Tracheal intubation	Therapeutic attitude	Endotracheal tube
Tracheostomy	Therapeutic attitude	Tracheostomy cannula
Bronchoscopy	Exam	Bronchofibroscopy
Non-invasive ventilation	Therapeutic attitude	Non-invasive ventilation
Cardiopulmonary resuscitation	General notes	
Placement of the ventilated patient in the prone position	Therapeutic attitude	Vental decubit
Introduction of nasogastric tube	Nursing intervention	Insert tube
	Therapeutic attitude	Nasogastric tube
Collection of respiratory samples by naso/ oropharyngeal swab		
Oropharyngeal aspiration of secretions or cough induction	Nursing intervention	Aspirate secretions
Respiratory kinesiotherapy	Nursing intervention	Perform respiratory kinesitherapy
Nebulizations	Doctor's prescription	
	Nursing intervention	Perform inhalation therapy

Table S2 – Complete analysis of patient and contextual characteristics and the risk of nosocomial SARS-CoV-2 (summarised in table 3 of the full text)

Table 3 -

	Probable (n=145)			Definitive (n=139)		
	**Crude analysis**	**M2[a]**	**M3[b]**	**Crude analysis**	**M2[a]**	**M3[b]**
**Age** (years)						
18-54	1			1	1	1
55-64	1.62 (0.47-5.65)	1.70 (0.48-5.97)	1.43 (0.37-5.55)	0.76 (0.2-2.87)	0.94 (0.24-3.68)	0.93 (0.24-3.64)
65-74	0.96 (0.27-3.43)	0.96 (0.27-3.46)	1.08 (0.28-4.08)	1.39 (0.49-3.96)	1.43 (0.49-4.18)	1.42 (0.49-4.15)
≥ 75	1.78 (0.60-5.21)	1.90 (0.60-6.04)	1.21 (0.35-4.14)	1.62 (0.54-4.90)	1.75 (0.56-5.45)	1.72 (0.55-5.38)
**Male**	0.94 (0.43-2.09)	0.96 (0.43-2.13)	1.38 (0.57-3.33)	0.85 (0.39-1.86)	0.85 (0.38-1.91)	0.86 (0.38-1.97)
**Urgent admission**	2.84 (0.79-10.15)	3.08 (0.82-11.55)	1.79 (0.43-7.37)	1.53 (0.53-4.39)	1.53 (0.51-4.62)	1.51 (0.50-4.58)
**Dependent patient**	1.79 (0.69-4.61)	1.82 (0.67-5.01)	1.34 (0.49-3.69)	0.87 (0.38-1.96)	0.86 (0.37-1.98)	0.88 (0.37-2.05)
**Comorbidities**	0.49 (0.20-1.19)	0.50 (0.20-1.24)	0.51 (0.20-1.30)	1.52 (0.51-4.52)	1.51 (0.50-4.53)	1.49 (0.49-4.50)
**Contextual characteristics[c]**						
**Emergency room visit**	3.51 (1.00-12.31)	3.87 (1.05-14.32)	2.94 (0.73-11.81)			
**Surgery**	0.25	0.24	0.25	1.03	1.1	1.11

	Probable (n=145)			Definitive (n=139)		
	Crude analysis	M2[a]	M3[b]	Crude analysis	M2[a]	M3[b]
	(0.05-1.23)	(0.05-1.16)	(0.05-1.26)	(0.41-2.62)	(0.42-2.9)	(0.42-2.91)
**Stay in non-refurbished wards**	**4.16 (1.59-10.85)**	**4.78 (1.65-13.83)**	**3.58 (1.18-10.87)**	1.27 (0.57-2.81)	1.14 (0.49-2.66)	0.72 (0.27-1.91)
**Number of different wards**						
1[c]	1	1	1	1	1	1
2	1.65 (0.68-4.02)	1.64 (0.66-4.07)	1.92 (0.73-5.00)	1.13 (0.44-2.91)	1.16 (0.43-3.12)	1.17 (0.44-3.13)
3	3.36 (0.87-13.02)	3.26 (0.84-12.73)	3.45 (0.81-14.73)	0.61 (0.18-2.13)	0.54 (0.16-1.91)	0.55 (0.16-1.96)
**Maximum number of beds in ward**						
1-4	1	1	1	1	1	1
5-9	1.95 (0.78-4.87)	2.12 (0.77-5.87)	1.06 (0.37-3.08)	2.12 (0.77-5.87)	1.27 (0.54-3.00)	1.40 (0.52-3.77)
≥10	2.09 (0.55-7.93)	2.02 (0.53-7.76)	1.65 (0.40-6.80)	2.02 (0.53-7.76)	0.21 (0.04-1.11)	0.21 (0.04-1.13)
**Cumulative duration of contact with other patients** (hours)						
≤750	1	1	1	1	1	1
751-1500	1.34 (0.55-3.28)	1.59 (0.57-4.44)	0.77 (0.24-2.49)	1.12 (0.45-2.79)	0.95 (0.37-2.43)	
>1500	0.70 (0.13-3.89)	0.87 (0.15-5.22)	0.52 (0.08-3.18)	0.6 (0.21-1.76)	0.46	0.75 (0.24-2.33)

	Probable (n=145)			Definitive (n=139)		
	**Crude analysis**	**M2[a]**	**M3[b]**	**Crude analysis**	**M2[a]**	**M3[b]**
					(0.14-1.51)	
**Roommate exposed to high risk procedures**	1.72 (0.69-4.32)	1.8 (0.63-5.15)	1.57 (0.51-4.86)	1.04 (0.48-2.25)	0.92 (0.4-2.11)	0.35 (0.09-1.41)
**Sharing room with SARS-CoV-2 positive patients already discharged from isolation**	**2.73 (1-7.48)**	**3.2 (1.03-9.92)**	2.51 (0.78-8.03)	2.44 (0.75-7.94)	2.23 (0.68-7.38)	2.27 (0.65-7.93)
**Sharing room with newly-diagnosed SARS-CoV-2 positive patients**	**3.84 (1.37-10.72)**	**3.85 (1.35-11.02)**	**3.35 (1.09-10.3)**	**10.17 (2.2-46.97)**	**9.91 (2.13-46.2)**	**9.92 (2.11-46.55)**

[a]After adjusting for matching variables; [b]After adjusting for matching variables and stay in non-refurbished wards; [c]Assessed in the 14 days prior to index date.

Table S3 Analysis restricted to patients in non-refurbished wards

	Probable	Definitive
Age (years)		
18-54	1	1
55-64	0.66 (0.09-5.06)	0.37 (0.02-6.8)
65-74	0.48 (0.08-2.83)	3.22 (0.51-20.23)
≥75	0.64 (0.13-3.12)	2.08 (0.34-12.59)
Male	1.83 (0.62-5.37)	1.47 (0.46-4.69)
Urgent	1.42 (0.09-23.14)	
Dependent patient	1.46 (0.34-6.31)	0.66 (0.16-2.79)
Comorbidities		
14 days prior to index date		
Emergency room stay[a]	3.39 (0.31-37.52)	
Duration of emergency room (ER) stay		
No ER stay	1	
ER stay ≤12 h	1.68 (0.36-7.73)	
ER stay >12 h	0.53 (0.11-2.6)	
Type of emergency room (ER)		
No ER stay	1	
ER Non-Covid-19 area	3.68 (0.33-41.25)	
ER-Covid-19 area	2.85 (0.23-36.01)	
Surgeries	0.20 (0.02-2.02)	3.97 (0.39-40.26)
Number of different wards		
1	1	1
2	5.91 (1.28-27.29)	0.54 (0.11-2.76)
3	12.99 (1.07-157.88)	0.21 (0.02-2.36)
Maximum number of beds in a ward		
≤4	1	
5-9	2.14 (0.56-8.2)	0 (0-0)
≥10	6.27 (0.63-62.21)	0 (0-0)
ROOMMATES		
Duration of contact (hours)		
≤750	1	1
751-1500	1.59 (0.57-4.44)	0.95 (0.37-2.43)
>1500	0.87 (0.15-5.22)	0.46 (0.14-1.51)
Roommate exposed to high risk procedures	1.24 (0.3-5.1)	1.34 (0.35-5.09)
Sharing room with SARS-CoV-2 positive patients already discharged from isolation	2.8 (0.72-10.95)	2.33 (0.45-11.91)
Sharing room with newly-diagnosed SARS-CoV-2 positive patients	2.92 (0.84-10.17)	2.27 (0.40-12.94)

[a]Only for probable cases and respective controls;

Table S4 – Analysis excluding patients who had been in intensive care in the previous 14 days

	Probable	Definitive
Age (years)		
18-54	1	
55-64	1.32 (0.26-6.68)	0.39 (0.06-2.6)
65-74	0.67 (0.14-3.17)	0.76 (0.2-2.86)
≥75	1.98 (0.53-7.41)	0.83 (0.22-3.08)
Male	2.65 (0.67-10.43)	1.16 (0.45-3.03)
Urgent	2.43 (0.78-7.52)	2.37 (0.57-9.94)
Dependent patient	2.43 (0.78-7.52)	1.59 (0.53-4.76)
Comorbidities	0.55 (0.2-1.49)	0.79 (0.17-3.58)
14 days prior to index date		
Emergency room stay[a]	2.84 (0.74-10.81)	
Duration of emergency room (ER) stay		
No ER stay	1	
ER stay ≤12 h	1.26 (0.27-5.94)	
ER stay >12 h	0.88 (0.21-3.75)	
Type of emergency room (ER)		
No ER stay	1	
ER Non-Covid-19 area	3.07 (0.77-12.17)	
ER-Covid-19 area	2.29 (0.45-11.74)	
Surgeries	0.13 (0.01-1.13)	0.98 (0.28-3.37)
Stay on an old ward	4.01 (1.16-13.83)	1.08 (0.37-3.11)
Number of different wards		
1	1	1
2	2.79 (0.98-7.92)	2.24 (0.67-7.5)
3	0 (0-0)	0 (0-0)
Maximum number of beds in a ward		
≤4		
5-9	2.36 (0.82-6.81)	1.14 (0.42-3.05)
≥10	0 (0-0)	0 (0-0)
ROOMMATES		
Duration of contact (hours)		
≤750	1	1
751-1500	2.64 (0.73-9.46)	0.77 (0.24-2.54)
>1500	0 (0-0)	0.4 (0.09-1.72)
Roommate exposed to high risk procedures	1.87 (0.55-6.39)	1.01 (0.36-2.88)
Sharing room with SARS-CoV-2 positive patients already discharged from isolation	7.69 (1.30-45.43)	1.95 (0.40-9.63)
Sharing room with newly-diagnosed SARS-CoV-2 positive patients	3.17 (0.81-12.38)	6.03 (1.22-29.78)

[a]Only for probable cases and respective controls;
